# Enabling Remote Patient Monitoring Through the Use of Smart Thermostat Data in Canada: Exploratory Study

**DOI:** 10.2196/21016

**Published:** 2020-11-20

**Authors:** Kirti Sundar Sahu, Arlene Oetomo, Plinio Pelegrini Morita

**Affiliations:** 1 School of Public Health and Health Systems University of Waterloo Waterloo, ON Canada; 2 Institute of Health Policy, Management, and Evaluation University of Toronto Toronto, ON Canada; 3 Department of Systems Design Engineering University of Waterloo Waterloo, ON Canada; 4 eHealth Innovation, Techna Institute University Health Network Toronto, ON Canada; 5 Research Institute for Aging University of Waterloo Waterloo, ON Canada

**Keywords:** disruptive technology, health information systems, public health surveillance, health behavior, Internet of Things, cell phone, mobile phone

## Abstract

**Background:**

Advances in technology have made the development of remote patient monitoring possible in recent years. However, there is still room for innovation in the types of technologies that are developed, used, and implemented. The smart thermostat solutions provided in this study can expand beyond typically defined features and be used for improved holistic health monitoring purposes.

**Objective:**

The aim of this study is to validate the hypothesis that remote motion sensors could be used to quantify and track an individual’s movements around the house. On the basis of our results, the next step would be to determine if using remote motion sensors could be a novel data collection method compared with the national census-level surveys administered by governmental bodies. The results will be used to inform a more extensive implementation study of similar smart home technologies to gather data for machine learning algorithms and to build upon pattern recognition and comprehensive health monitoring.

**Methods:**

We conducted a pilot study with a sample size of 8 to validate the use of remote motion sensors to quantify movement in the house. A large database containing data from smart home thermostats was analyzed to compare the following indicators; sleep, physical activity, and sedentary behavior. These indicators were developed by the Public Health Agency of Canada and are collected through traditional survey methods.

**Results:**

The results showed a significant Spearman rank correlation coefficient of 0.8 (*P<*.001), which indicates a positive linear association between the total number of sensors activated and the total number of indoor steps traveled by study participants. In addition, the indicators of sleep, physical activity, and sedentary behavior were all found to be highly comparable with those attained by the Public Health Agency of Canada.

**Conclusions:**

The findings demonstrate that remote motion sensors data from a smart thermostat solution are a viable option when compared with traditional survey data collection methods for health data collection and are also a form of zero-effort technology that can be used to monitor the activity levels and nature of activity of occupants within the home.

## Introduction

### Background

Remote patient monitoring (RPM) involves digital technologies used to collect and transmit medical and health data from individuals to health care providers for assessments and recommendations [1,2]. As a component of public health surveillance, novel RPM technology infrastructures can modernize traditional, time-consuming, and expensive data collection methods [1]. The Internet of Things (IoT) enables health care providers to remotely monitor patient health and analyze the data with minimal delay to develop a personalized treatment plan or follow the patient’s progress over time [3]. Aspects that can be monitored include physical activity levels, drug adherence, and physiological indicators such as blood pressure and heart rate [1,4]. The ultimate goal of RPM is to enable individuals to lead healthier lives and improve their well-being at home by leveraging technology.

Although a variety of RPM platforms exist today [5], consumers continue to be limited by interoperability issues because the systems lack compatibility, often creating silos of technologies where data cannot be integrated or exchanged. This study uses existing off-the-shelf technologies, such as IoT-based smart thermostats equipped with wireless motion sensors and consumer-level wearable fitness trackers. Smart Wi-Fi thermostats regulate indoor air temperature via motion sensors that detect occupancy. Their benefits include cost and energy savings owing to increased heating and cooling efficiency and their ability to eliminate the need for an additional dedicated home monitoring platform. In the United States, customers using these smart thermostats save approximately 23% on heating and cooling costs [6]. For this study, the Ubiquitous Health Technology Lab (UbiLab) [7] at the University of Waterloo has partnered with ecobee, a Toronto-based Canadian smart Wi-Fi thermostat company [8]. Using data from ecobee thermostats and a variety of sensors, the UbiLab Public Health Surveillance Platform (UPHSP) was developed to augment current public health surveillance efforts through the use of IoT data. Public health surveillance is labor intensive, requires significant human resources, often relies on outdated data, and has numerous types of biases (eg, recall, performance, nonresponse, voluntary response, social desirability) [9]. The UPHSP improves that scenario by providing access to near real time, objective sensor-based data.

Public health surveillance is the systematic collection, analysis, and interpretation of health data that are required to determine funding for programs, strategies, and initiatives [10]. It is also an important component of public health that focuses on early identification, mitigation of disease, prevention, planning, and overall evaluation of population health behaviors. At present, the Canadian public health surveillance system relies on self-reporting and short-term activity monitoring to collect data on health indicators included in the Physical Activity, Sedentary Behavior and Sleep (PASS) Indicator framework [11]. This method has implications for research methodology, the validity of research results, and the soundness of public policy developed from evidence using questionnaire-based research [12]. Traditional methods have several limitations. First, they are prone to recall and social desirability bias. Second, data collection does not occur in real time and requires extensive administrative resources adding to the total costs [13]. Third, conducting surveys can be a lengthy process and thus are done periodically and intermittently. Finally, methods such as physical activity monitoring require participants to be completely involved in data collection, which may be inconvenient and cause deviations from their regular routines, leading to data collection challenges that result in smaller study sizes [14].

### Objectives

The goal of this study is to develop and facilitate the use of UPHSP across Canada for population- and individual-level surveillance of health behaviors. The platform enables RPM via smart Wi-Fi thermostats and motion sensors to seamlessly collect and transmit health behavior data. These data are provided to public health officials, care providers (with minimum disturbance of daily activities and delivery of personalized health insights), and users. UPHSP has advantages over conventional data collection methods because it addresses current challenges with traditional data collection techniques. UPHSP allows researchers to access granular and longitudinal data generated directly from within the participant’s house. The anonymized data will be collected 24 hours a day and 7 days a week and then consolidated so that they are available to health care officials in near real time. The core strength of this surveillance method is that it utilizes zero-effort technology (ZET) [14] that requires no effort from users. This effectively reduces the burden on study participants, ensures minimal disturbance to their daily routines, and can also be applied in the real-world environment [15].

In collaboration with the Public Health Agency of Canada, UbiLab is developing UPHSP to improve and develop individual- and population-level health behavior indicators. In the initial stages of the project, the team focused on the existing set of health indicators from the PASS Indicators framework. PASS Indicators measured from within the house were selected for this study. This study was then submitted to the Healthy Behaviour Data Challenge organized by the Public Health Agency of Canada (PHAC), the Canadian Institutes for Health Research, and the MaRS Discovery District in August 2017 [16,17]. The aim was to demonstrate that collecting reliable data for the 3 indicators was possible with smart home technology. The goal is to enable public health officials, such as the PHAC, to access real time population-level data of Canadian behaviors and develop policies and programs with relevant data to improve the health and well-being of Canadians.

## Methods

**Figure 1:**
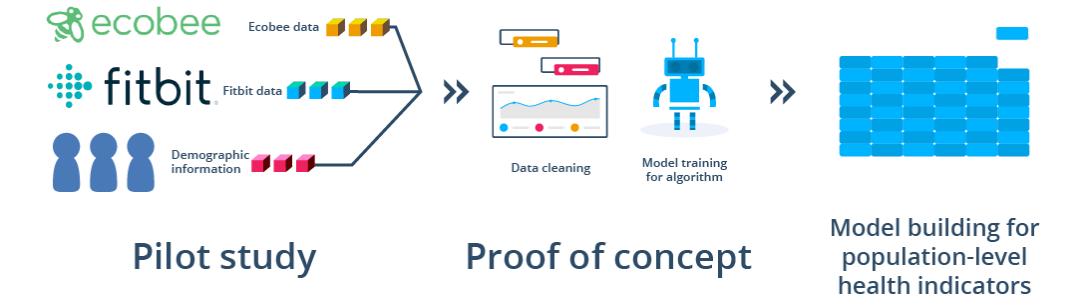
The process of model building for population-level health surveillance.

UPHSP is based on thermostat sensor technology that collects raw motion data generated by activity in the house. A two-step approach was carried out to achieve the objectives of this study. The first step involved deploying a small pilot study to validate the accuracy of the motion sensors, and the second step investigated the larger data set acquired via the ecobee’s Donate Your Data (DYD) program [18,19] to demonstrate the scalability of this study, as described in Figure 1.

### Pilot Study

**Figure 2:**
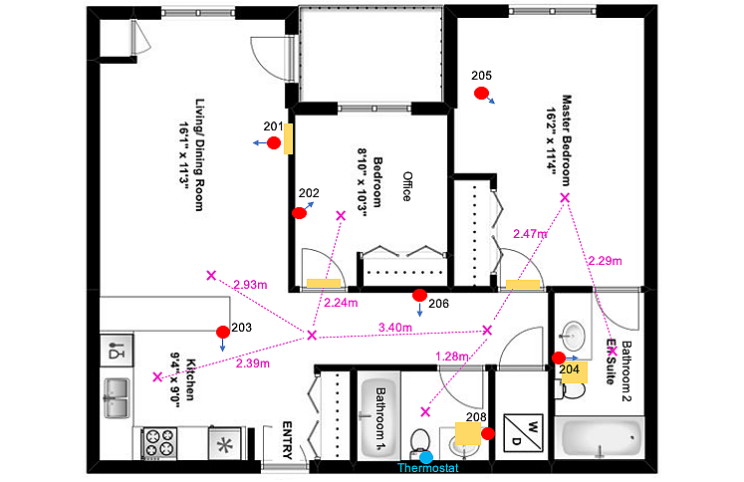
Sample home layout of a participant with ecobee thermostat and remote sensors.

The first important task for this study was to determine if the ecobee sensors were a valid data source. The association between remote motion sensors and a person’s movement in the house needed to be definite and accurate. A pilot study was conducted to validate the relationship between ecobee remote sensor activation and movement within the house and to test the accuracy of these sensors. The team hypothesized that there would be a significant association between the number of sensors activated and the number of steps taken by the participants in the house. The study included a total of 8 participants (4 women and 4 men) aged between 25 and 41 years and were residents of the Kitchener-Waterloo Region in Ontario, Canada. Each participant wore a Fitbit Zip and was the sole occupant in their house during the data collection period. Furthermore, each house was equipped with an ecobee thermostat unit, and remote sensors were placed around the house to ensure maximum coverage by the sensors as seen in the Figure 2. Approximately 5 to 30 remote motion sensors were deployed in each home, depending on the size of the house. The floorplan layout for each house was obtained to determine optimum sensor placement. Motion and step data were collected in all 5 houses via the thermostat sensors and the Fitbit between 9:00 AM and 5:00 PM. Participants were also instructed to record their activities at all times and identify interruptions, such as using the washroom or getting a snack, in an activity log.

Once the data were collected, cleaned, and ready for analysis, the team was able to identify the activity status of the participants (active vs sedentary) using step data and remote sensor activations (Figure 1). As the data were not normally distributed, the Spearman correlation test was the tool of choice to test for association [20]. The Spearman correlation coefficient was found to be 0.8, with *P*<.001 indicating that there was a strong positive linear association between the total number of sensors activated and the total number of steps traveled by the participant within the house and that this correlation was statistically significant. The strength of this correlation increased with the increasing number of sensors within a larger house. With our first hypothesis successfully proven, the second phase of the project, as identified in Figure 1, could begin.

### DYD

Ecobee created the *Donate Your Data* (DYD) [21,22] program to provide researchers access to anonymized data collected from their technology for further energy and sustainability research. UbiLab, since its launch in 2016, is the first research team to use these data for health purposes and has gained access to a large data repository through this research partnership. The first data set received from ecobee contained over 10,000 records (or households) and has since increased to more than 110,000 records after 3 years.

The metadata information accompanying the thermostat motion data is provided by the ecobee user upon initial setup of their ecobee thermostat, where they may choose to opt into the program to contribute their data to the DYD program. The information that is collected includes the style of house (eg, apartment, townhouse, etc), floor square footage, number of floors, and number of occupants. Incomplete fields were eliminated from the analysis pool. For ecobee to maintain this program’s opt-in process and encourage participation, leaving the fields optional increased the enrollment but did not contribute to completeness or quality of the data set. The DYD data set is completely anonymized to maintain user privacy, and all user customizations of sensor names or locations are not shared with researchers. This is a limitation but a sensible move from ecobee to protect user privacy and encourage program participation. Unfortunately, the metadata quality is restricted by the amount and quality of the information entered during setup by the user.

From January 2015 to March 2017, data from 556 single occupant houses in the United States and 70 in Canada were included in the analysis. According to ecobee, over 1 million smart thermostats are installed in houses across the United States and Canada and that number continues to grow. Furthermore, federal and provincial rebate programs encourage user adoption of smart thermostat technologies [23]. These efforts are beneficial for this study and have future potential in UPHSP.

### A Comparison

The team focused on the ability to collect data from sensors and demonstrate how the same indicators could be measured with sensor technology. Existing health indicators from the PASS Indicator framework were matched on the basis of the measure description determined by the PHAC [11]. At present, the sleep indicator is a self-reported value determined by the Canadian Health Measures Survey (CHMS) from Statistics Canada and is the sum of the average number of hours spent sleeping in a 24-hour period by adults aged 18 to 79 years [24]. This is potentially problematic because the value is subject to recall bias and sleep times are rounded to the nearest half hour. In addition, the platform provides insight into the sleep timing, patterns, and number of disturbances captured owing to excessive movement or additional triggering of sensors in areas of the house other than the bedroom. Indicators of sleep interruptions would provide insights into sleep-related conditions and disorders such as sleep apnea and sleepwalking, but they have not yet been developed by the PHAC. However, the PHAC is currently developing several new sleep indicators for sleep quality, sleep hygiene, and more.

Adherence to the PASS Indicators of physical activity guidelines and the total amount of moderate-to-vigorous physical activity is derived from CHMS and uses accelerometer data [25]. As the platform functions within the house, the lack of movement during the day very likely indicates that the individual was out of the house. Canada has distinct seasons; therefore, this information could be used to investigate seasonal patterns that contribute to social isolation or increased healthy behavior during the warmer months.

### Physical Activity

Ecobee data are streamed in 5-min intervals, which means that every 5 min the remote sensors send the motion data to the thermostat and the thermostat sends these data to the server. Hence, all the analyses performed in the project were performed in 5-min intervals. The total activity within the house was measured for the purposes of our study. Physical activity was defined as true if ≥2 sensors were activated at 5-min intervals within the house during the waking period between 8:00 AM and 10:00 PM. This method of measuring physical activity provides real time data, removes the involvement of the user from data collection, effectively eliminates recall bias, and minimizes the social desirability effect.

### Sedentary Behavior

Sedentary behavior was defined as less than 2 sensors being activated within any 5-min interval during the day (between 8:00 AM and 10:00 PM) while the individual was in the house. As this was a pilot study, we tried to classify unknown data into 3 categories: sleep, sedentary behavior, and physical activity. Our assumptions were as follows: (1) zero sensor activation was labeled as sleep; (2) 3 or more activated sensors indicated physical activity; and (3) the remaining values indicated sedentary behavior. The logic behind this type of sampling was that if an individual moved from one place to another within 5 min, at least two sensors would be activated and the energy equivalent used would be less than when undertaking physical activity. When more than 2 sensors were activated, the step values were more appropriately deemed to be a higher level of physical activity.

This is different from the PHAC accelerometer-generated indicator obtained from CHMS [26] because the focus was on sedentary time within the house as our technology did not measure sedentary time spent commuting or while at work. The algorithms developed for the project treated movement between rooms as physical activity and only stagnant behavior was considered sedentary.

## Results

### Pilot Study

Initially, a pilot study was conducted to confirm the accuracy of the responses of the ecobee sensors to movement in the house. It involved using Fitbit to track the steps of 8 participants for approximately one week, which was equivalent to 386 person-hours of data.

**Figure 3:**
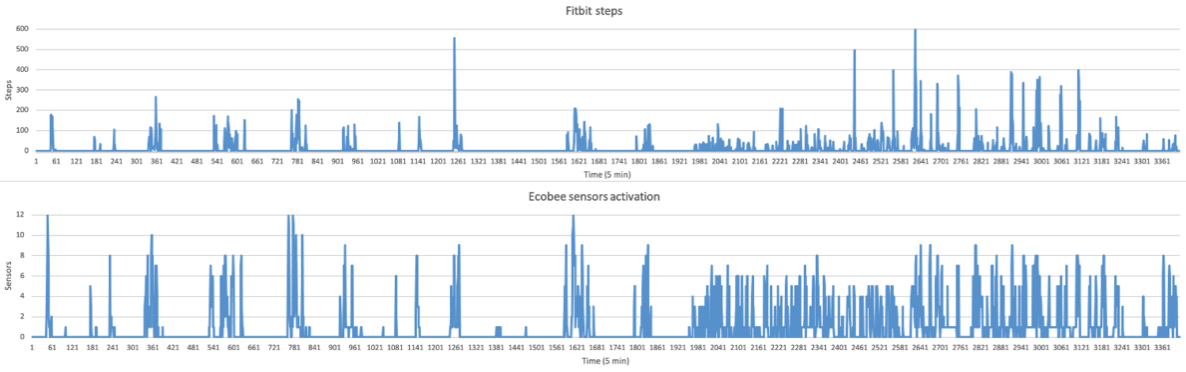
Association between Fitbit and ecobee database–separated.

**Figure 4:**
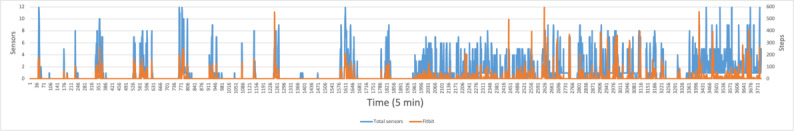
Association between Fitbit and ecobee database–superimposed.

**Figure 5:**
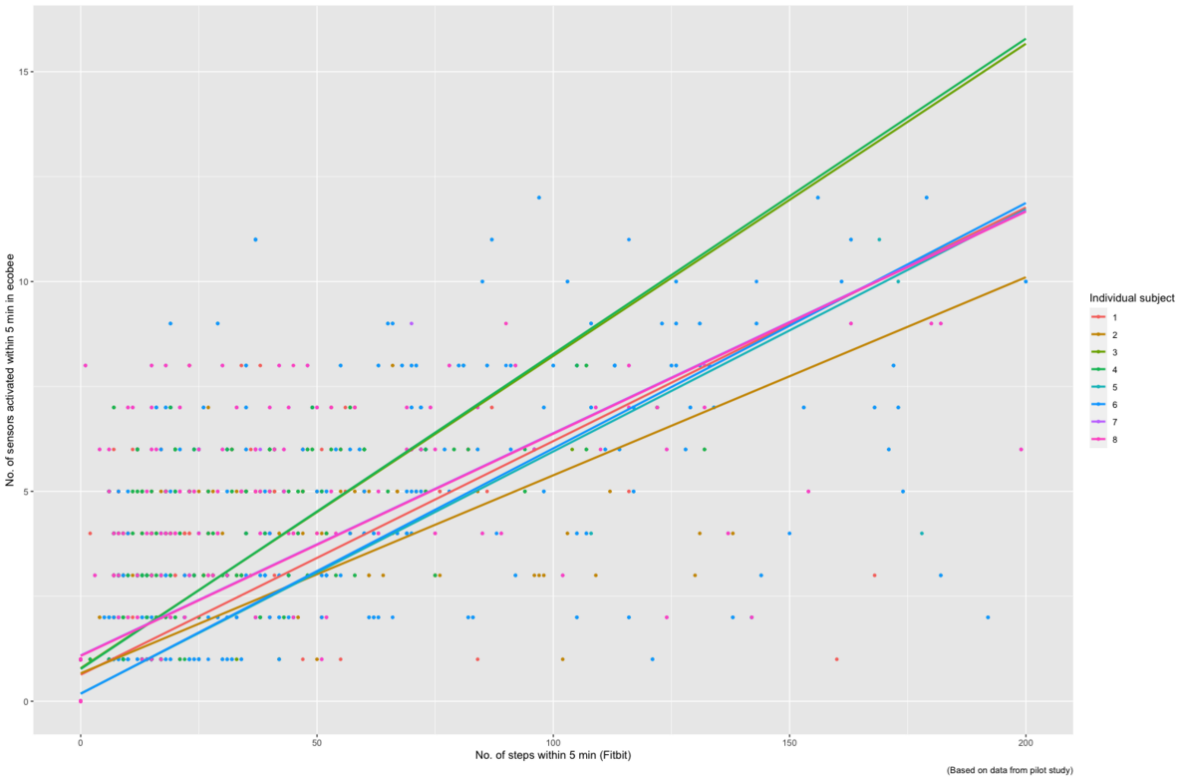
Scatter plot with individual correlation.

A total of 8 participants used Fitbit for 1 week; however, after processing and cleaning the data, not all of the data were exactly of same length across the 7 days. There was no significant difference between the duration of participants’ data used. A simple visual inspection of the number of steps captured by the Fitbits and sensor activations was done to determine alignment (as seen in Figures 3 and 4). There is a clear increase in motion sensor activation in alignment with increased steps taken. To confirm that the sensors were working precisely, a statistical test was performed to measure the association between the sensor activation and Fitbit data. The Spearman correlation coefficient was r=0.8 (range 0.78-0.90; n=3292; *P*<.001). These results indicate a strong association between sensor activation and the number of steps recorded via Fitbit. Figures 3 and 4 illustrate the sensors that were activated when an individual walked around their house and their association with steps taken by that individual captured via Fitbit. The scatterplot in Figure 5 demonstrates the data points and individual-level correlation between steps and activated ecobee sensors. In addition, traveling between rooms, which is indicated by more steps, showed higher levels of activity on the sensors. During periods of inactivity, when no steps were recorded by Fitbit, the sensors also displayed no or minimal activation. A few random or unexplained activations may be caused by internal movements of items in the house, such as a moving curtain or a bird flying by a window that faced a sensor. These movements were considered noise and removed for the purposes of our analysis. Only one sensor was activated during this time, which can be attributed to disturbed sleep or other kinds of sedentary behavior.

### Sleep

Following the pilot study, the second phase of the project involved analyzing the larger DYD data set from ecobee. Sleep in households was measured using UPHSP. In Table 1, the individual and household columns are defined as follows: a house with a single occupant was labeled as *individual* and households with more than one occupant was labeled as *household*. They were mutually exclusive. The findings were that, on average, single occupant households from the ecobee data set had a sleep duration of 7.8 hours per day. Compared with the conclusions from previous surveys and PHAC’s current method of measuring average Canadian sleep duration [24], the platform’s result was 0.7 hours higher on average. UPHSP can calculate the number of sleep interruptions during the night using automated algorithms. Data recorded by ecobee revealed approximately two hours of interrupted sleep during regular sleep by ecobee single occupancy residents. An interruption was defined as any movement within the sleeping hours. Interrupted sleep is a new indicator as it is not currently possible to self-report this measure and has not been collected or reported by the PHAC.

**Table 1:** Key findings and a comparison of UbiLab findings with PHAC reported physical activity, sedentary behavior, and sleep indicators (n=958).

Name of indicators	UbiLab^a^		PASS^b^
	Individual (n=70)	Household (n=888)	Individual
Nighttime sleep, hours	7.89	7.71	7.2
Disturbed sleep, hours	2.10	2.28	N/A^c^
Physical activity in the house, minutes per day	85.2	146.4	24.1
Sedentary time, hours	4.44	5.75	9.6
Away period, hours	8.12	5.80	N/A

^a^UbiLab: Ubiquitous Health Technology Lab.

^b^PASS: physical activity, sedentary behavior, and sleep.

^c^N/A: not applicable.

### Physical Activity

The second indicator measured by UPHSP was the amount of physical activity in each household, which averaged 85 min per day. Physical activity was defined as movement within the house during waking hours. A comparison of our indicator with physical activity (moderate to vigorous) measurements reported by the PHAC revealed that UPHSP results surpassed that of PHAC by almost an hour (85 min vs 24 min). However, our measurements do not consider the intensity of the physical activity (ie, moderate or vigorous). Further studies are required to develop intelligent algorithms that can differentiate between the intensities of physical activity.

Analyzing the data over a long period can inform public health officials about seasonal physical activity variations. During the winter months, the number of indoor activities and the time spent inside will likely increase owing to cold weather and shorter days. Measuring household physical activity in Canada is therefore essential and necessary from a public health perspective.

### Sedentary Behavior

The study participants recorded their activity log during the pilot study to track the type and duration of their activities. The platform measures general activities in the house, time patterns of doing certain types of housework, the number of interruptions, and the area of the house where the users spend most of their time. The log depicts the total time spent on a certain activity that is recorded by a participant. In the first row, the time the user spent cleaning their house would be considered physical activity as the user was active and moved around the house. In this case, multiple sensors would have been active throughout the house during a 5-min interval. The second row indicates that the user was sitting and watching television. This would be considered a sedentary behavior if less than 2 sensors were activated in a 5-min interval. The number of interruptions is a new indicator that could help the PHAC understand healthy behaviors and seasonal patterns.

UPHSP calculates the true nature and timing of all the behaviors being carried out in the house. In addition, the data set was filtered for any exceptional behavior to ensure that there was an accurate analysis of the ecobee users’ average activities. For this purpose, 100 steps (156 m) were selected as the threshold for a 5-min interval. If participants took more than 100 steps in a 5-min interval (eg, while working out on a treadmill), these occurrences were considered outliers. To select this set point of 100 steps, the Spearman correlation statistical test was performed on several thresholds beginning with 100 steps, which was increased by 50 steps at a time to a maximum of 400 steps. As seen in the Multimedia Appendix 1, there was only a small amount of variation in the correlation coefficients, r≥0.798 and *P*<.001, at 100 steps, which indicates a strong correlation between the distance traveled and the activation of sensors. Therefore, the project was successfully able to use ecobee remote sensors to understand indoor physical activity levels.

## Discussion

### Principal Findings

Technological advancements have rapidly become cross-disciplinary as they merge health care and medicine. However, unlike the retail and commercial sectors, the health care field lags behind [25]. Mobile health is the application of digital technology for the use of medical care and has the goal of empowering individuals to be in control of their own health. Technology has revolutionized the field of medicine and continues to impact all areas of health care. Medical technology has evolved rapidly, enabling doctors to use new equipment inside hospitals and allowing them to connect with patients and other physicians thousands of kilometers away through smart devices [27]. Hundreds of health and wellness mobile apps have been developed since the introduction of smartphones (also known as the field of mobile health [mHealth]) [28]. These apps enable users to track their own health (ie, fitness levels, water consumption, diet, etc) and compare their data with standardized guidelines or challenge their peers to step competitions. The dependence on medical technology cannot be ignored within the health care industry because health care professionals can continue to find ways to improve their practice with the development of these brilliant innovations [27].

Manipulating the temperature of a house using a smartphone was not possible 10 years ago. At present, monitoring health and diagnosing conditions are possible via smart home technologies. With this technology, UbiLab is turning valuable sources of data into useful insights. Raw data are difficult to interpret and would be insufficient to encourage behavioral change. They are made interpretable with artificial intelligence (AI) algorithms using data from the pilot study. Interpreted health data will be shared with users using mobile or web-based platforms and will be presented through a dashboard. These data could help individuals learn about their daily activity patterns, general health behaviors, and compare their behaviors with those of their peers or the national average. For this to become a reality, it is necessary to ensure that all the users can view and interpret the data anywhere with clarity. A simple dashboard interface will be carefully designed to display meaningful and actionable health information for Canadians. The team created a simple dashboard for the purpose of the Healthy Behaviour Data Challenge. Ideally, such a dashboard would display the user’s health data in a textual, auditory, and visual manner. It would support multiple languages and also be interactive and visually appealing to gain more attention from individuals. Furthermore, the dashboard would recommend changes to the user’s physical activity, sleep, and sedentary behaviors to improve their overall health. Smart home technologies are currently expanding into the realm of virtual assistants that are based on AI such as Amazon Echo [29], Apple’s Siri [30], and Google Home [31]. Incorporation of these technologies could bring additional personalization to the platform and user interaction and further motivate health behavior change and enhance user experience.

UPHSP has shown potential by providing a possible improvement and by increasing the efficiency of the current labor-intensive methods of data collection. In comparison with traditional data collection methods that require labor, funding, and ample time, this platform is more cost-effective because it leverages existing technology (smart thermostats). The platform is very convenient for most users because it uses ZET and only requires direct involvement for the initial setup and orientation of the system. In addition, the data collected via UPHSP is more granular and accurate, collected 24/7 in real time, which eliminates potential social desirability and recall bias. On average, the current delay in research and policy development is 17 years [27,32,33], which can be changed with the help of technology and multisite studies. Implementing UPHSP could significantly reduce the time spent on data entry and collection because the technology requires little active involvement from study participants and can reduce the overall study time.

The implementation of this study is possible because of a growing interest in smart home technology. An estimated 100,000 Canadian households have an ecobee thermostat installed, with even more users in the United States of America, and this number is only expected to increase. Incentives from the provincial government, such as the Ontario Green Fund, has also helped drive interest and adoption. Similar initiatives would diversify the sample population, which is valuable as it would open up this technology to a wider sociodemographic of population. The issue of a biased sample population can be addressed by investing in the adoption of smart thermostat across the country. By including individuals from different cultures, ages, and various socioeconomic statuses, UPHSP can help the PHAC gain insight into contrasting health behaviors among different segments of the country. This could help identify populations with the greatest need and inform policies that target these groups.

There are many potential applications of this type of technology and ways of leveraging of nontraditional data sources, as has been done here with data from IoT for public health surveillance [34]. For example, public health officials can monitor risky behavior for chronic diseases, and this could have a significant impact on routine monitoring systems at the national and provincial levels. Real time monitoring of population health behaviors without interfering with daily activity is the biggest strength of this study. As technology rapidly evolves to become more affordable, smaller, and wireless, passive and seamless data collection for health monitoring becomes more feasible.

### Limitations

The team has identified some limitations with the technology that constrain the capabilities of the data collected and the possible health behavior insights. The ecobee remote sensors collect data at 5-min intervals, which from the perspective of collecting data for temperature control is sufficient. However, a higher level of granularity is required for RPM, and the system is not yet able to differentiate between multiple, different occupants in a house. There is no means to tag an individual to identify them as they move through the house; therefore, observing a pattern is difficult, and it is not yet possible to attach it to a specific person. Identifying or differentiating occupants in the house is currently challenging without adding an additional on-body device. The larger DYD data set is a biased sample because the typical buyer of the smart thermostat is a middle-to-upper class individual who is interested in cost and energy savings, reducing environmental impact, and technologically savvy. Initiatives from governments and smart technology manufacturers to encourage the adoption of smart thermostat technologies, such as rebates or the installation of this technology in affordable housing units, can help to make this more accessible to a wider income range and reduce the sample bias. The distribution will also need to adequately reflect the Canadian population, and considerations should be taken to include smaller or remote communities outside of the provinces of Ontario and British Columbia.

Moving forward, the UbiLab will collect demographic information from a subset of existing ecobee users to understand the association between age, sex, and other relevant demographic indicators. Our plan is to explore other sensor technologies to train machine learning algorithms and generate data. In addition, solutions to correctly identify true positive presence in the house will be explored to address this shortcoming.

### Conclusions

There is a lot of potential for RPM to expand and leverage commercial technologies. This study is just one example where technology can be used to bring innovative solutions for real use in the realm of health care, especially as it allows the use of technologies that are zero effort and have more than one added benefit. Technologies such as these will be able to advance the fields of RPM and public health surveillance.

## Appendix

Multimedia Appendix 1 Spearman correlation coefficients for each of the data cleaning stages.

## Abbreviations

AI: artificial intelligence
CHMS: Canadian Health Measures Survey
DYD: Donate Your Data
IoT: Internet of Things
PASS: Physical Activity, Sedentary Behavior and Sleep
PHAC: Public Health Agency of Canada
RPM: remote patient monitoring
UbiLab: Ubiquitous Health Technology Lab
UPHSP: UbiLab Public Health Surveillance Platform
ZET: zero-effort technology
