# Supplementary material for: Enabling Remote Patient Monitoring Through the Use of Smart Thermostat Data in Canada: Exploratory Study
Source: JMIR Mhealth Uhealth. 2020 Nov 20;8(11):e21016. doi: 10.2196/21016 (PMC7718086; doi:10.2196/21016)
Supplement: Multimedia Appendix 1 [file mhealth_v8i11e21016_app1.docx]

## Appendix – Spearman correlation coefficients for each of the data cleaning stages

| Filter used | Number of rows | Person hours | Pearson correlation coefficient | P value |
| --- | --- | --- | --- | --- |
| All clean data | 3412 | 386.3 | 0.5512 | <.001 |
| Less than 100 steps only | 3292 | 274.3 | 0.798 |  |
| Less than 150 steps only | 3352 | 279.3 | 0.774 |  |
| Less than 200 steps only | 3374 | 281.1 | 0.766 |  |
| Less than 300 steps only- | 3392 | 282.6 | 0.709 |  |
| Less than 400 steps only | 3408 | 284.0 | 0.600 |  |
